# Treatment of Knee Osteochondral Lesions Using a Novel Clot of Autologous Plasma Rich in Growth Factors Mixed with Healthy Hyaline Cartilage Chips and Intra-Articular Injection of PRGF

**DOI:** 10.1155/2017/8284548

**Published:** 2017-07-17

**Authors:** Ramón Cugat, Eduard Alentorn-Geli, Gilbert Steinbacher, Pedro Álvarez-Díaz, Xavier Cuscó, Roberto Seijas, David Barastegui, Jordi Navarro, Patricia Laiz, Montserrat García-Balletbó

**Affiliations:** ^1^Fundación García-Cugat, Barcelona, Spain; ^2^Mutualitat Catalana de Futbolistes, Federación Española de Fútbol, Barcelona, Spain; ^3^Artroscopia GC, Hospital Quirón, Barcelona, Spain; ^4^Universitat Internacional de Catalunya, Barcelona, Spain

## Abstract

Knee cartilage or osteochondral lesions are common and challenging injuries. To date, most symptomatic lesions warrant surgical treatment. We present two cases of patients with knee osteochondral defects treated with a one-step surgical procedure consisting of an autologous-based matrix composed of healthy hyaline cartilage chips, mixed plasma poor-rich in platelets clot, and plasma rich in growth factors (PRGF). Both patients returned to playing soccer at the preinjury activity level and demonstrated excellent defect filling in both magnetic resonance imaging and second-look arthroscopy (in one of them). The use of a clot of autologous plasma poor in platelets with healthy hyaline cartilage chips and intra-articular injection of plasma rich in platelets is an effective, easy, and cheap option to treat knee cartilage injuries in young and athletic patients.

## 1. Introduction

Chondral or osteochondral lesions are common disorders of the knee and are on the rise due to the ageing of the population and the current active lifestyle. Due to their poor healing and regenerative potential, injuries involving the hyaline cartilage are challenging to treat, particularly in the young and athletic population. Cartilage defects may lead to early-onset osteoarthritic processes and a tremendous impairment in the function and quality of life in these patients. Symptomatic injuries usually require surgical treatment.

Knee cartilage injuries may be treated by chondroplasty, microfractures, mosaicplasty (osteochondral autograft transfer), osteochondral allograft transplantation, scaffold-based repair (with or without cell therapy), autologous chondrocyte implantation (ACI), or matrix-induced ACI (MACI) [1–4]. Some pitfalls of these techniques include fibrocartilage formation without long-lasting improvement (especially in high-level athletes), expensive treatments, long recovery time, or unpredictable results in the athletic population.

To understand PRP-based therapies is fundamental to understanding the platelet biology. The platelets have a major role in haemostasis, inflammation, and proliferation for remodeling and healing tissue and also have an angiogenic power to deliver molecules into the damaged tissue.

The purpose of this study was to report the outcomes of a one-step surgical procedure consisting in a clot of autologous mixed plasma poor-rich in platelets with healthy hyaline cartilage chips and intra-articular injection of plasma rich in platelets (PRP) to treat knee osteochondral lesions (KOL).

## 2. Case Presentation

### 2.1. Case  1

**Figure 1:**
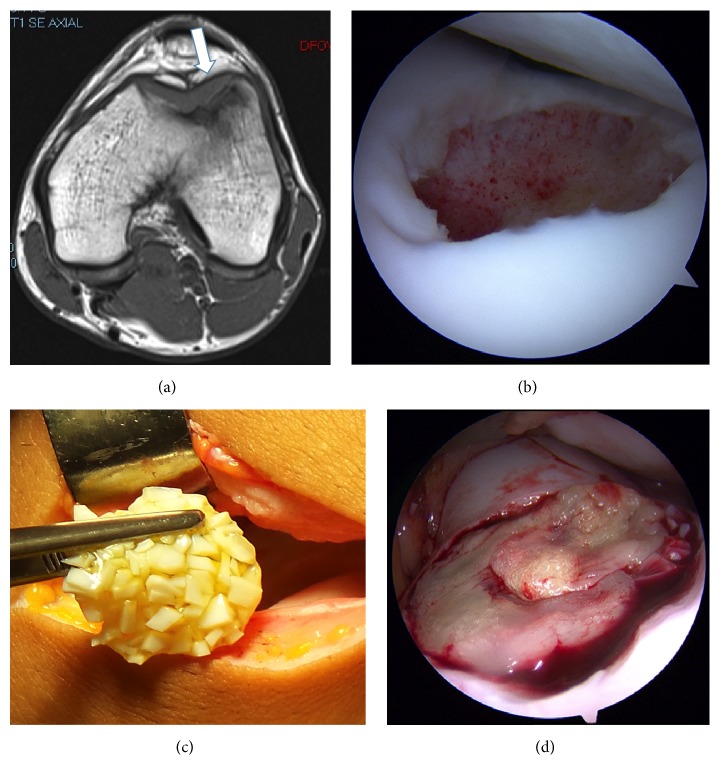
Composition of figures demonstrating the initial injury and surgical treatment. (a) Preoperative axial T1-sequence MRI demonstrating the osteochondral injury (white arrow). (b) Intraoperative picture demonstrating the debrided defect in the lateral femoral condyle. (c) Intraoperative picture demonstrating the autologous matrix before implantation. (d) Arthroscopic view of the filled defect at the end of the procedure.

A 16-year-old male soccer player was referred to our service complaining of left knee pain. The pain was worse with running and both concentric and eccentric contractions. On physical exam, he had pain to palpation in the inferior pole of the patella, no effusion, no meniscal lesions, good stability, and normal range of motion. He was initially diagnosed of patellar apophysitis and a course of physical therapy started. The patient did not improve and a magnetic resonance imaging (MRI) was requested at 3 months, demonstrating an osteochondritis dissecans of the lateral femoral condyle at the level of the trochlea of 20 × 18 mm diameter and 8 mm depth (Figure 1(a)). Surgical treatment was recommended. The basal functional scores of the patient were Lequesne index 4 (in a 0 to 24 scale where 0 is no functional limitation) [5], visual analogue scale for pain 6 (in a 0 to 10 scale where 0 is no pain), WOMAC 11/96 (11.5%), IKDC Subjective Knee Evaluation Form 75.9, and Tegner-Lysholm 88.

Knee arthroscopy was performed five days later and a KOL was confirmed (Figure 1(b)). Samples of healthy-looking hyaline cartilage in the defect edges were obtained using a curette. Hyaline cartilage was cut up until several chips were obtained. A vertical rim of cartilage wall was left in the defect edges. A debridement of the lesion was carried out with a shaver down to the subchondral bone. Then, a 3 cm longitudinal arthrotomy was performed and the knee moved into a position that could completely expose the KOL.

PRGF preparation was conducted using the Endoret^©^ PRGF^©^ system (BTI Biotechnology Institute, Álava, Spain). Eighty mL of blood was extracted before surgery and placed in eight 9 ml tubes containing 3.8% of a citrate solution. A BTI System IV^©^ (BTI Biotechnology Institute, Álava, Spain) centrifuge was used for 8 minutes at 580*g* obtaining the sedimentation of red and white cells at the bottom and platelets with plasma on the top part of the tubes. After centrifugation, there were two differentiated fractions. The first fraction, in the upper part of the supernatant, was plasma poor in platelets (PPP), which was placed in BTI 9 ml, sterile-fractionation tubes. The second fraction (just over the white cells layer) was the plasma rich in platelets (PRP) placed in other BTI 9 ml, sterile-fractionation tubes. Extraction of the second fraction is a critical step and must be done carefully to avoid aspiration of white cells.

To prepare the clot, a 50/50 mixture of first fraction (PPP) along with second fraction (PRP) is activated with CaCl_2_ following the rate of 0.02 ml per milliliter of plasma, maintaining the tube at room temperature. Cartilage chips were combined with the mixed PPP-PRP and left for at least 30 minutes until a semisolid matrix was formed (Figure 1(c)). Time to form the matrix will depend on each patient blood. Then this matrix was placed in the KOL, which has to be slightly below the healthy surrounding articular cartilage to avoid overgrowth (Figure 1(d)). Great care was taken to ensure adequate and homogesneous sitting of the matrix in the defect, and after 5 minutes, gentle range of motion under direct visualization was performed to ensure adequate stability of the construct. Next, the arthrotomy and portals were closed and the second fraction was injected once intra-articularly after platelet activation with calcium chloride. Two cast splints were used to immobilize the lower extremity for two weeks.

After a week, the patient had no pain at all. After the cast splints were removed, gentle range of motion was allowed from 0° to 90°, progressing at 15° per week afterwards. Non-weight-bearing was kept for 4 weeks and then partial weight-bearing was allowed from weeks 4 to 6. Full weight-bearing started at 6 weeks after surgery. Strengthening exercises were resumed at 8 weeks. An MRI was requested at two months, which demonstrated adequate filling and incorporation of the matrix in the defect without overgrowth. At 5 months, running and unrestricted weight training were allowed. A follow-up MRI was obtained at 6 months, which demonstrated complete filling of the defect by chondrocyte-like fibrous tissue without maturation. At 8 months, he returned to play at the same preinjury level.

**Figure 2:**
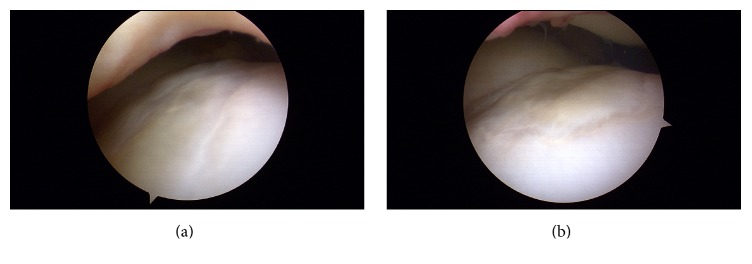
Two views of the second-look arthroscopy. ((a) and (b)) Intra-articular view of the femoral trochlea demonstrating the formed cartilage flush with the surrounding cartilage, adequate filling of the defect, and adequate transition between the healthy and formed cartilage.

The patient was doing very well, but one year after surgery he had an anterior cruciate ligament (ACL) injury while playing with the national soccer team. The MRI demonstrated the ACL tear but the KOL appeared intact and, in fact, the defect was almost completely restored. ACL reconstruction was conducted using a bone-patellar tendon-bone autograft using the single-bundle anatomic technique. This gave us the opportunity for a second-look arthroscopy of the KOL. The defect was completely filled with the new tissue, which had a similar color and consistency (Figure 2(a)) and was smooth compared to the surrounding healthy cartilage (Figure 2(b)). The patient is now at 6 months post-op from the ACL reconstruction and is near completion of the readaptation phase to competition. At a total of 20 months after the index procedure (but only 6 months after the ACL reconstruction) the patient had the following scores: SF-36 67 (physical function 95, physical role 25, pain 77.5, general health 95, vitality 65, social function 65, emotional role 66.6, mental health 64, and health transition 50), Lequesne index 0 (in a 0 to 24 scale where 0 is no functional limitation) [5], visual analogue scale for pain 0.5 (in a 0 to 10 scale where 0 is no pain), WOMAC 2/96 (2.1%), IKDC Subjective Knee Evaluation Form 90, and Tegner-Lysholm 100.

### 2.2. Case  2

**Figure 3:**
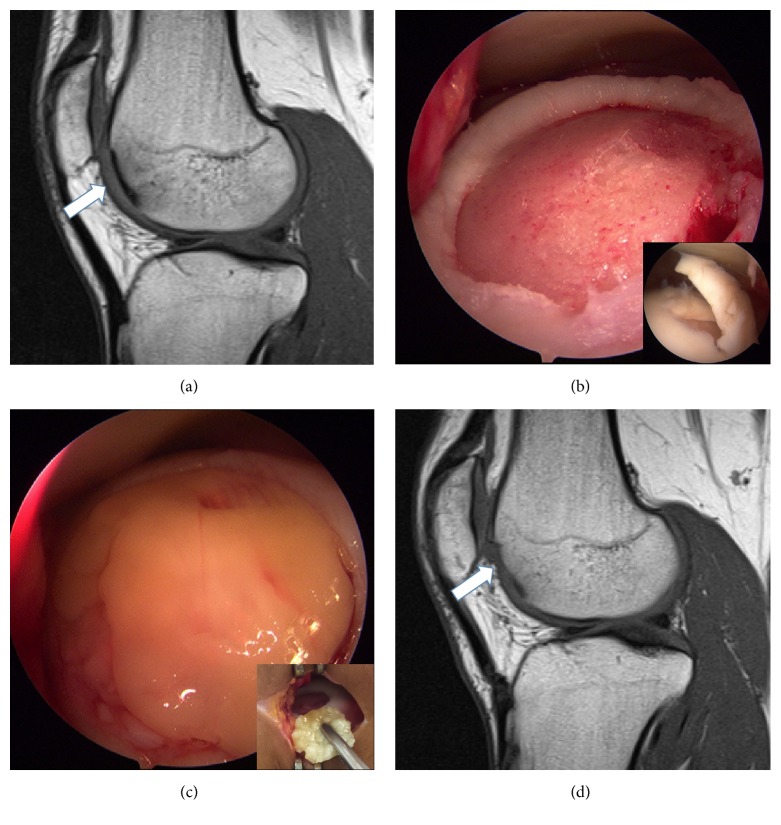
Composition of figures demonstrating the initial injury, surgical treatment, and postoperative MRI 6 months after surgery. (a) Preoperative sagittal T1-sequence MRI demonstrating an osteochondral injury (white arrow). (b) Intraoperative picture demonstrating the debrided defect in the lateral femoral condyle. The small picture shows the injury seen during the diagnostic arthroscopy. (c) Intraoperative picture demonstrating the filled defect after the autologous matrix had been applied. (d) Postoperative sagittal T1-sequence MRI demonstrating adequate filling and incorporation of the matrix in the lateral femoral condyle and absence of subchondral oedema (white arrow).

A 22-year-old male soccer player came to our clinic complaining of knee pain, swelling, and inability to play soccer without pain. Physical exam revealed pain in the lateral femoral condyle, mild joint effusion, and full range of motion. An MRI demonstrated a grade IV osteochondral injury in the lateral femoral condyle. The patient was initially treated nonoperatively and returned to playing soccer at his preinjury level. However, at 1.5 years from the first visit the patient had a painful forced valgus maneuver, no joint effusion, a stable knee, and range of motion, but inability to play soccer pain-free. The MRI showed the same osteochondral injury and bone oedema in the lateral femoral condyle (Figure 3(a)). Due to lack of improvement with 1.5 years of physical therapy and activity modification, an arthroscopy was recommended to evaluate and treat the KOL. The basal functional scores of the patient were Lequesne index 3 (in a 0 to 24 scale where 0 is no functional limitation) [5], visual analogue scale for pain 5 (in a 0 to 10 scale where 0 is no pain), WOMAC 8/96 (8.3%), IKDC Subjective Knee Evaluation Form 46, and Tegner-Lysholm 60.

Knee arthroscopy demonstrated a rounded 1.7 cm diameter osteochondral injury in the lateral femoral condyle (Figure 3(b)). The surgical procedure and postoperative protocol detailed above were repeated for this patient (Figure 3(c)). As in case 1, the patient had no pain after one week of the operation and this was maintained throughout the postoperative period. At two months, the patient had full range of motion and a follow-up MRI was requested, demonstrating good filling and incorporation of the matrix into the defect. At three months after surgery, the patient had slight joint effusion and quadriceps atrophy. At six months, he still had significant quadriceps tone asymmetry and a follow-up MRI demonstrated an almost complete filling of the defect without still complete maturation but with no other significant associated lesions (Figure 3(d)). The patient slowly recovered quadriceps tone, and at eight months he was allowed to begin running. One year after surgery, the MRI showed adequate repair of the defect with correct thickness (no overgrowth) and similar signal intensity in the defect compared to surrounding healthy articular cartilage. The patient returned to playing at the same preinjury level 10 months after the surgical procedure. At 20 months after surgery, the patient had the following scores: SF-36 96.4 (physical function 100, physical role 100, pain 100, general health 100, vitality 80, social function 100, emotional role 100, mental health 88, and health transition 100), Lequesne index 0 (in a 0 to 24 scale where 0 is no functional limitation) [5], visual analogue scale for pain 0 (in a 0 to 10 scale where 0 is no pain), WOMAC 0/96 (0%), IKDC Subjective Knee Evaluation Form 100, and Tegner-Lysholm 100.

## 3. Discussion

Cartilage repair is very difficult to achieve due to the impossibility for the chondrocyte to migrate through the dense matrix, the absence of mesenchymal cells within the cartilage, and absence of blood vessels. For these reasons, superficial lesions have limited-to-inexistent regeneration potential, while extensive full-thickness injuries that involve subchondral bone can theoretically initiate physiological repair and regeneration because of blood supply in the subchondral bone that triggers the regeneration. However, regeneration is uncommon and the lesion undergoes a repair response where fibrocartilage with inferior biomechanical properties is formed within the defect.

The treatment of symptomatic KOL includes chondroplasty, microfractures, mosaicplasty (osteochondral autograft transfer), osteochondral allograft transplantation, scaffold-based repair (with or without cell therapy), or autologous chondrocyte implantation (ACI) [1–4]. Chondroplasty is a palliative procedure with suboptimal results and likely no potential to modify the progression to osteoarthritis. Therefore, despite this is a very easy, fast, and nonaggressive procedure, it should not be first option to treat athletes with full-thickness cartilage defects or osteochondral injuries. Microfracture is a very popular procedure to treat KOL. In fact, this is a simple, cheap, and usually fast-recovering operation with good results and return to sports rate [6]. However, the procedure creates a fibrocartilage with inferior biologic and biomechanical properties, which likely explains the potential worse long-term outcomes compared to cartilage regenerative techniques [4]. Osteochondral transplantation procedures may provide good long-term outcomes and return to sports rate [6, 7]. However, autograft transplantation is limited to smaller defects, and allografts are limited to donor availability and both have higher costs compared to microfracture. Scaffold-based and ACI procedures are good cartilage regenerative procedures but there is still lack of good quality studies with long-term follow-up, especially in the athletic population [4]. In addition, these are very expensive procedures (cultures also require two surgical procedures) that may not be available for many people or medical centers.

Sánchez et al. have reported successful outcomes in a patient with articular cartilage avulsion treated with plasma rich in growth factors and biodegradable pins [8]. The use of a clot of autologous mixed PPP-PRP with healthy hyaline cartilage chips and intra-articular injection of PRP is a novel procedure for KOL that may have several advantages over other procedures: (1) cheap procedure (no use of graft transplantation instrumentation, scaffolds, or chondrocyte cultures); (2) easy and fast procedure using a small arthrotomy; and (3) no rejection possibility because the patients' own tissue, cells, and plasma are used (safe procedure). An important aspect is that this procedure has similar return to sports rate and time to return compared to the other procedures [6].

Interestingly, case one had a new injury with enough energy to tear the ACL, but the regenerated cartilage previously repaired resisted the twisting movement and it actually had an excellent appearance and consistency, with a normal hyaline-like cartilage. Both patients did very well clinically and by imaging studies, with return to play at the preinjury activity level.

This study has some limitations. First, this is a report of two cases with relatively limited follow-up length. Second, to avoid the invasive procedure, no biopsies have been taken from the formed tissue to obtain a histology evaluation of the defect-filling tissue. Third, no comparative group with other surgical options or rehabilitation is provided in this study.

## 4. Conclusion

The use of a one-step surgical procedure consisting of a clot of autologous mixed PPP-PRP with healthy hyaline cartilage chips and intra-articular injection of PRP is an easy, effective, safe (patient's own products), reproducible, and cheap alternative option to treat osteochondral injuries of the knee in young and athletic patients. However, this is only a preliminary report involving two cases and, therefore, subsequent studies with larger sample size and even a comparative group should be conducted to confirm or refute these promising results.
